# Perioperative Teaching and Feedback: How are we doing in Canadian OTL-HNS programs?

**DOI:** 10.1186/s40463-019-0330-2

**Published:** 2019-01-17

**Authors:** Z. Chaudhry, M. Campagna-Vaillancourt, M. Husein, R. Varshney, K. Roth, A. Gooi, LHP Nguyen

**Affiliations:** 1 Department of Medicine, McGill University, Montreal, Canada; 2 Department of Otolaryngology – Head and Neck Surgery, McGill University, 1001 Decarie Boulevard, Room A02-3015, Montreal, Quebec H4A 3J1 Canada; 3 Department of Otolaryngology – Head and Neck Surgery, Western University, London, Canada; 4 Department of Otolaryngology – Head and Neck Surgery, University of Manitoba, Winnipeg, Canada; 5 Centre for Medical Education, McGill University, Montreal, Canada

**Keywords:** Feedback, Perioperative, Teaching, Education, Otolaryngology.

## Abstract

**Background:**

Discrepancies between resident and faculty perceptions regarding optimal teaching and feedback during surgery are well known but these differences have not yet been described in Otolaryngology - Head and Neck Surgery (OTL-HNS). The objectives were thus to compare faculty and resident perceptions of perioperative teaching and feedback in OTL-HNS residency programs across Canada with the aim of highlighting potential areas for improvement.

**Methods:**

An anonymous electronic questionnaire was distributed to residents and teaching faculty in OTL-HNS across Canada with additional paper copies distributed at four institutions. Surveys consisted of ratings on a 5-point Likert scale and open-ended questions. Responses among groups were analysed with the Wilcoxon-Mann Whitney test, while thematic analysis was used for the open-ended questions.

**Results:**

A total of 143 teaching faculty and residents responded with statistically significant differences on 11 out of 25 variables. Namely, faculty reported higher rates of pre and intra-operative teaching compared to resident reports. Faculty also felt they gave adequate feedback on residents’ strengths and technical skills contrary to what the residents thought. Both groups did agree however that pre-operative discussion is not consistently done, nor is feedback consistently given or sought.

**Conclusion:**

Faculty and residents in OTL-HNS residency programs disagree on the frequency and optimal timing of peri-operative teaching and feedback. This difference in perception emphasizes the need for a more structured approach to feedback delivery including explicitly stating when feedback is being given, and the overall need for better communication between residents and staff.

## Background

The development of technical skills and the acquisition of surgical decision making skills are fundamental competencies for all graduating Otolaryngology –Head and Neck Surgery (OTL-HNS) residents, yet studies have estimated that, on average, surgical residents spend only 6–14% of their total working hours in the operating room (OR) [1]. Furthermore, the quality of operative experience is influenced by the interactions between residents and faculty, and thus varies significantly between individuals [2].

Surgical specialties are challenged further by pressure for reduced operative expenses and increased productivity, unique patient safety considerations, and other factors that may influence the educational experience of residents in the operating room [3]. Thus, with the current state of restricted training hours and the recent move to competency-based medical education, there exists a pressing need for improving the educational experience of OTL-HNS residents in the operating room. [4].

Traditionally, the educational process has centered mainly around the concepts of teaching and assessment. However, an integral part of the learning cycle is the feedback provided by teachers, which serves to highlight specific improvement points that can be used by students to close the learning gap. Feedback is a formative tool that ensures learning is *informed*, with the ultimate goal of improving future performance [5]. As evidence of its importance, the Royal College of Physicians and Surgeons of Canada has incorporated feedback within the core scholar competency of the CanMEDS framework. Despite this, studies examining the administration of operative feedback have shown that residents are typically unsatisfied with the quantity and quality of feedback received [1, 4, 6, 7].

Although the benefits of teaching and feedback have been well proven [1], discrepancies exist regarding the perceptions of perioperative education, the optimal preparation for surgery, and intraoperative teaching methods and feedback [8]. The current study sought to evaluate perceptions of perioperative teaching and feedback among residents and teaching faculty in OTL-HNS across Canada.

## Methods

Institutional ethics board approval at McGill University (Montreal, Canada) was granted prior to the start of the study. The aim of this study is to compare faculty and resident perceptions of perioperative teaching and feedback in OTL-HNS residency programs across Canada with the goal of highlighting potential areas for improvement.

### Survey development

Two surveys, one targeted to residents and the other to teaching faculty, eliciting opinions on teaching style and feedback, as well as pre-operative and intra-operative teaching and feedback, in OTL-HNS were created. Surveys were modified versions of validated instruments in research in surgery [4] and obstetrics and gynecology [7] that were modified via content experts and experts in medical education. Surveys consisted of open-ended questions as well as ratings on a five-point Likert scale, with “*5*” representing “*consistently or always*”, “*3*” being “*about half the time*” and “*1*” indicating “*rarely or never*”. Residents and faculty were asked to self-report demographic information (university, age, gender, PGY-level, years of experience). As well, both groups were asked to rate the frequency with which pre-operative teaching occurred (anatomy review, case review, potential pitfalls discussed, expectations of residents discussed, and specific instructions given to residents). Residents and faculty were also asked to grade how likely it was that residents were asked which specific skills they wanted to practice in the OR, if residents were permitted to perform critical steps of the surgery, as well as if there was a supportive environment in the OR, among others. Residents and faculty were asked about the types of feedback given as well as the optimal method and timing of feedback. Open-ended questions regarding the qualities of an ideal teacher and weaknesses among teachers were asked.

### Study population

The study population included all Canadian OTL-HNS residents and faculty from academic teaching institutions. Teaching faculty was defined as OTL-HNS surgeons involved in regular operative teaching of OTL-HNS residents and having at least one operative day per month within a teaching institution. OTL-HNS residents that had not completed a minimum of six months of dedicated OTL-HNS service were excluded. Eligibility for inclusion in the study was met based on self-reported data from faculty and residents completing the survey.

### Survey distribution

An anonymous electronic questionnaire was distributed electronically to the Canadian Society of OTL-HNS mailing list in April, June and September 2015. Additional paper copies were distributed at four university institutions to increase the response rate among residents and faculty. Paper copies were distributed at grand rounds over a three-month period at the University of Manitoba, Western University, McGill University and Université de Montréal. Appropriate consent was obtained from all participants.

### Data analysis

Survey responses among groups were analysed with the two-tailed t-test. Means were calculated for resident and faculty responses across each variable, and statistical significance was determined as a < 0.05 difference between the two groups. Teaching or feedback was considered done well if the mean response from residents and faculty was ≥3.5 and mean responses < 3.5 described practices that were considered poorly done. The 3.5 value was arbitrarily chosen in order to define concordance as more than half the time, to ensure that something that was considered “done well” truly was so and not in fact representing a neutral response. Thematic analysis was used for qualitative open-ended questions.

## Results

### Demographic characteristics

A total of 77 faculty members and 66 residents from 13 institutions responded to the questionnaire. Response rates were highest among faculty with over 20 years of work experience as well as among 5th year residents. Response rates could not be calculated, as the exact number of academic faculty in Canada is unknown. Demographic data for both groups are found in Table 1.

**Table 1:** Demographics of residents and teaching faculty

	RESIDENT	FACULTY
	*n* (%)	*n* (%)
Gender		
• Male	37 (62.7%)	64 (84.2%)
• Female	22 (37.3%)	12 (15.8%)
PGY Level		
• PGY 1	15 (23.8%)	
• PGY 2	10 (15.9%)	
• PGY 3	7 (11.1%)	N/A
• PGY 4	10 (15.9%)	
• PGY 5	16 (25.4%)	
• Fellow	5 (7.9%)	
Years as attending staff		
• 0–5		16 (20.3%)
• 6–10	N/A	14 (17.7%)
• 11–15		14 (17.7%)
• 16–20		9 (11.4%)
• > 20		26 (32.9%)
Province		
• Nova Scotia	3 (5.1%)	5 (6.6%)
• Quebec	17 (28.8%)	19 (25.0%)
• Ontario	15 (25.4%)	19 (25.0%)
• Manitoba	4 (6.8%)	9 (11.8%)
• Alberta	4 (6.8%)	8 (10.5%)
• British Columbia	1 (1.7%)	9 (11.8%)
• Other	0 (0%)	2 (2.6%)
• Not specified	15 (25.4%)	5 (6.6%)

The results of the survey are summarized in Tables 2, 3 and 4. Statistically significant differences were found on 11 variables, marked by a red asterisk in the tables, with four differences pertaining to feedback and seven pertaining to teaching. Faculty and residents did not significantly differ on the remaining 14 variables. Note that, despite certain variables showing statistically significant differences, faculty and residents may have still agreed on whether the variable was being done well or poorly, and thus differed merely on the *extent* to which those things were done well or poorly.

**Table 2:** Congruity between faculty and residents on “DONE WELL” teaching and feedback objectives

AGREE AND DONE WELL	RESIDENT mean ± SD	FACULTY mean ± SD	95% CI
TEACHING			
Graduated learner autonomy from one case to the next	3.8 ± 1.1	4.2 ± 0.8	0.0, 0.7
Builds confidence in resident skills	4.0 ± 0.8	4.4 ± 0.8	0.1, 0.7
Supportive learning environment	3.9 ± 1.0	4.2 ± 0.7	0.0, 0.6
FEEDBACK			
Constructive and non-judgmental	3.8 ± 0.7	4.1 ± 0.7	−0.0, 0.5
Faculty consistently provide feedback during surgery	3.5 ± 0.9	4.0 ± 0.9	0.2, 0.8
Important immediately after operation	4.0 ± 0.9	4.0 ± 0.9	−0.3, 0.3

**Table 3:** Congruity between faculty and residents on "DONE POORLY" teaching and feedback objectives

AGREE AND DONE POORLY	RESIDENT mean ± SD	FACULTY mean ± SD	95% CI
TEACHING			
Review the anatomy	2.3 ± 0.9	3.0 ± 1.1	0.4, 1.1*
Express expectations of residents	2.6 ± 1.1	3.4 ± 1.2	0.5, 1.3*
Takes over case if inadequate pace	3.5 ± 1.0	3.4 ± 1.0	−0.5, 0.2
Asks which specific skills want to practice	2.4 ± 0.9	2.9 ± 1.2	0.2, 0.9*
Allows external pressures (ex: time, nursing) to influence teaching	3.4 ± 1.1	3.2 ± 1.1	−0.5, 0.2
FEEDBACK			
Provide examples of weaknesses	3.2 ± 0.9	3.3 ± 0.9	−0.2, 0.5
On operative decision making	2.8 ± 1.0	3.4 ± 0.9	0.2, 0.9*
Residents ask for feedback on surgical skills	2.8 ± 1.0	2.3 ± 0.8	−0.9, − 0.2*
Residents feel comfortable giving feedback to faculty on their teaching skills	2.2 ± 1.1	2.7 ± 1.1	0.1, 1.0*
Faculty ask for feedback on teaching skills	1.6 ± 0.9	2.0 ± 1.0	0.1, 0.8
Faculty explicitly inform residents when they are giving feedback	2.7 ± 0.9	3.0 ± 1.1	−0.1, 0.6

**Table 4:** Incongruity between faculty and residents teaching and feedback objectives

DISAGREE	RESIDENT mean ± SD	FACULTY mean ± SD	95% CI
TEACHING			
Review the case	3.0 ± 1.1	3.8 ± 1.0	0.5, 1.2*
Review the planned procedure	3.2 ± 1.1	3.8 ± 1.0	0.3, 1.0*
Discuss potential pitfalls of the surgery	2.9 ± 1.0	3.8 ± 1.0	0.5, 1.2*
Provide clear instructions about case	3.1 ± 1.0	4.2 ± 0.8	0.7, 1.4*
Encourages residents to verbalize their thought process	3.4 ± 1.1	3.7 ± 1.0	−0.0, 0.7
Resident performs critical steps	3.3 ± 1.0	3.6 ± 1.0	−0.0, 0.6
FEEDBACK			
On technical skills	3.4 ± 0.7	3.8 ± 0.8	0.1, 0.7
Provide examples of strengths	2.9 ± 0.8	3.7 ± 0.8	0.5, 1.1*

### “Done well” - concordance between resident and faculty perceptions

Faculty and residents agreed that teachers, allowed for graduated learner autonomy in the OR, built confidence in resident skills, created a supporting learning environment, provided constructive feedback, and provided feedback during the OR (Table 2). Both groups also agreed that it is important to receive feedback immediately after the OR.

### “Done poorly “- concordance between resident and faculty perceptions

Faculty and residents recognized specific areas for improvement regarding perioperative teaching: namely the influence of external pressures on teaching, if faculty took over the case due to inadequate pace, pre-operative teaching of anatomy, faculty expressing their expectations of residents, and discussing skills residents wanted to practice in the OR (Table 3). In terms of feedback, both groups agreed that the following were done poorly: residents initiating the request for feedback, faculty explicitly saying when they were providing feedback, faculty asking for feedback on their teaching skills, residents giving faculty feedback on teaching skills, faculty providing residents with examples of their weaknesses, and on resident operative decision-making skills.

### Discordance between resident and faculty perceptions

Regarding teaching, faculty reported that the following points were done well while residents disagreed: faculty reviewed the case and planned procedure with the resident preoperatively, gave clear instructions to the resident about the case prior to the OR, discussed common procedural pitfalls, allowed residents to perform critical steps of the surgery, and encouraged residents to verbalize their thought process in the OR (Table 4). Regarding feedback, faculty again perceived the following variables to be well executed while residents did not: faculty provided feedback on residents’ strengths and technical skills.

### Open ended questions

When asked to list the attributes of “ideal” and “non-ideal” teachers, both faculty and residents agreed on overall themes of 1) patience (“*Patient - instead of taking over, providing clear specific instructions on how to work faster or more efficiently*.”), 2) provision of graded autonomy (“*Progressively allows more control to be taken by the learner - demonstrates the steps in a stepwise fashion allowing the learner to gain more experience and take more control each time.”),* and 3) creation of a supportive environment (“Encouragement in moment: When [residents] get “stuck” [faculty] do not simply take over but instruct how to get back on course”) (Table 5). Residents, when compared to faculty, more strongly emphasized feedback as a key attribute: “[Faculty] Calmly give constructive feedback,” “I like a teacher who is able to identify my weaknesses, but who is also able to tell me how to improve it”. Faculty emphasized the non-ideal trait of being overly critical towards residents.

**Table 5:** Thematic analysis of open-ended questions regarding ideal and non-ideal attributes of teachings

ATTRIBUTES OF TEACHERS	RESIDENT (%)	FACULTY (%)	REPRESENTATIVE QUOTE
IDEAL	*n* = 42	*n* = 55	
Provides feedback	48	20	*“Combination of positive and negative feedback given”*
Allows graded autonomy	29	25	*“Acting simply as an assist to allow you to act / think as primary surgeon”*
Demonstrates patience	38	38	*“Allows the resident time to think, and enough time to attempt a part of a surgery”*
Supportive	20	22	*“Allows residents to push themselves to develop independence and comfort”*
NOT IDEAL	*n* = 36	*n* = 46	
Impatient	42	28	*“Impatience if case going slowly”*
Takes over too quickly	36	28	*“Takes over from the resident too quickly without allowing them to work to a solution and thus learn”*
Inadequate or untimely feedback	33	17	*“Non-specific, delayed feedback”*
Intimidating/Overly Critical	17	30	*“Critical in front of other OR staff”*

As well, faculty valued the importance of teaching residents and having a teacher that is also a competent surgeon. They recognized weaknesses in education as there being a lack of teaching in the OR and poor time management in the OR. Additionally, faculty identified the greatest challenges to teaching and feedback in the OR as being time constraints and resident factors such as lack of preparation for the OR, lack of resident motivation, as well as the complexity of cases. While residents felt that the biggest challenges to teaching and feedback in the OR were time constraints, the burden of clinical responsibilities between cases, as well as limited time to prepare before the OR.

Linear regression models run on the data to determine whether or not there were differences in perception between residents and faculty based on gender, academic rank of faculty, number of days spent operating with residents, whether or not faculty have training in teaching and feedback, resident training level, and resident educational background were inconclusive.

## Discussion

To our knowledge, this is the first study to evaluate the perceptions of perioperative teaching and feedback among faculty and residents in OTL-HNS programs yielding several major findings. Recognizing the differences in perception will allow optimization of learning opportunities in a postgraduate curriculum that is transitioning to competency by design (CBD) for which OTL-HNS is one of the leaders.

Given the importance of maintaining anonymity and encouraging honest answers, we purposely chose to not have program or province specific data made extractable. Eliciting personal information about teaching and feedback from both faculty and residents is still a difficult subject for some to verbalize, especially for residents who may feel intimidated to respond a certain way. We thus wanted to make sure we removed that component for respondents to answer as freely as possible.

### Congruity between faculty and residents on “DONE WELL” objectives

Both faculty staff members and residents agreed that the teaching faculty allowed for graduated learner autonomy in the OR, built confidence in resident skills, and created a supportive learning environment (Table 2). Each of these skills are instrumental in CBD based curricula whereby faculty act as coaches to residents improving their skills through learning opportunities [9].

Faculty also did well in providing feedback during surgeries. This feedback skill involves short, focused and timely feedback delivery to residents and contributes to the successful implementation of CBD [9]. In addition, both groups agreed that it is important to receive feedback immediately after surgeries.

### Congruity between faculty and residents on “DONE POORLY” objectives

Faculty and residents agreed that more can be achieved regarding pre-operative teaching (Table 3). Studies have alluded to the natural history of the disease and surgery outcomes as the focus of faculty whereas the resident’s focus pertains to the technical aspects of the procedure [10, 11]. Solutions to this teaching gap may include setting clear expectations of pre-operative preparation [1] along with the incorporation of Entrustable Learning Activities (EPAs) in the new competency based curriculum. This will require explicit direction for residents as well as clear learning and teaching goals with the ultimate aim of assessing and improving learning [9].

Both groups recognized that feedback was both inconsistently given and sought. This can be possibly due to resident’s failure in recognizing learning opportunities [12], language barriers [1] and differing definitions of feedback [13]. Jensen et al. suggested that by explicitly informing residents that feedback is being given by saying: “I am giving you feedback”, there is less ambiguity regarding feedback [4]. Another feedback issue is the informality by which it is given particularly in fast-paced environments, thus not meeting the needs of the learner [14]. Cox et al. asserted that the cause of limitations in feedback delivery is likely due to the lack of formal training of surgeons as educators [15], usually attaining these skills by observation and self-reflection of their own teaching experiences [16, 17]. Solutions to this issue include faculty development and mentor workshops, faculty development teaching fellowships, as well as graduate programs in medical education [16]. In addition, the Royal College has developed evidence based assessment templates to assist clinicians in documenting work-based assessments (WBAs) guiding faculty in delivering focused and specific feedback [9].

Faculty and residents recognized that feedback is not consistently being sought. Research has shown that individuals tend avoid information that is detrimental to their self-image [18]. Another obstacle to feedback seeking is the perception that it reflects incompetence [19]. Feedback seeking is important as it can positively affect the learners adaptability, performance and overall learning [20]. Moreover, the new post graduate curriculum WBAs will be implemented and require multiple teaching observations that can both be initiated by residents and faculty [9]. Solutions to this issue include alternative methods of feedback through documentation as individuals are less likely to seek feedback when in a public setting [20, 21]. Also, previous studies have demonstrated that a quality relationship between trainees and supervisors increased the likelihood among learners to seek negative feedback [22]. Thus training leaders to encourage feedback seeking behavior could ultimately improve the resident’s educational experience [20].

### Perception INCONGRUITY between faculty and residents

Finally, both groups disagreed on certain aspects pertaining to faculty skills. More specifically, faculty believed they scored well on giving feedback on strengths and technical skills whereas residents felt these were not adequately met (Table 4). Feedback in CBD emphasizes recognizing and praising a resident’s strengths as a means of continued reinforcement [9]. Cox et al. emphasize the importance of faculty’s self-reflection on their own teaching skills and suggest the implementation of regular resident-based assessments of teaching to improve feedback from academic surgeons [15], which has been validated in other studies [23]. Moreover, when observed over time, faculty have demonstrated improved performance as perceived by residents in response to suggestions for improvement [24].

To appreciate the importance of feedback, we have to look no further than the CBD curricula which are being unfolded throughout Canadian residency programs, with OTL-HNS being one of the first. This study however has illustrated some of the existing hurdles that participants will face during the deployment of these programs. Some of the strategies that may be employed to counter these could include faculty training on how to give feedback using simulation based teaching. Another critical component to moving forward will be to create a culture where residents feel safe and empowered to ask for feedback. Further studies will be necessary to evaluate this data as it is likely to evolve with the implementation of CBD curricula, as are the strategies to cope with these discrepancies.

### Limitations

Limitations in our study include low response rates among faculty and residents. In our study, more faculty completed the questionnaires compared to residents. In addition, those who are dissatisfied with the quality and quantity of feedback may have been more likely to complete the survey, thus making the study results less generalizable. Results are also reflective of subjective measures of feedback as there are no objective measures of its administration. Finally, the results are subject to recall bias as the surveys were administered at grand rounds and electronically and were not necessarily completed immediately after a day in the OR.

## Conclusion

Feedback forms an integral component of medical education and, in fact, so much so that whole new curricula of CBD are being implemented to reflect this. Although residents and faculty agree on the importance of feedback, the relative reported frequencies of feedback vary. While intra-operative teaching is done well, there is a lack of pre-operative teaching and an absence of feedback administration and seeking behaviors. These gaps in teaching can be detrimental to the learning experience of residents who face restricted training hours in the OR with increasingly demanding clinical schedules. Although there are limitations to our study, the results obtained are consistent with those obtained across analogous studies conducted in other disciplines. The results emphasize the need for more formalized feedback administration and training in teaching and feedback among both faculty and residents. Strategies to improve the discrepancies demonstrated by this study are necessary to maintain quality surgical education for residents. Further studies are needed to evaluate their effectiveness particularly in light of the change to a competency-based curriculum.

## Abbreviations

OR: Operating Room
OTL-HNS: Otolaryngology - Head and Neck Surgery
WBAs: work-based assessments
